# Supplementary material for: The causal role of circulating inflammatory markers in osteoporosis: a bidirectional Mendelian randomized study
Source: Front Immunol. 2024 Jul 18;15:1412298. doi: 10.3389/fimmu.2024.1412298 (PMC11291241; doi:10.3389/fimmu.2024.1412298)
Supplement: Supplementary file 1 [file DataSheet_1.zip › supplementary material/CIM-OSTEOPOROSIS /GCST90274804.csv_leave_one_out.pdf]

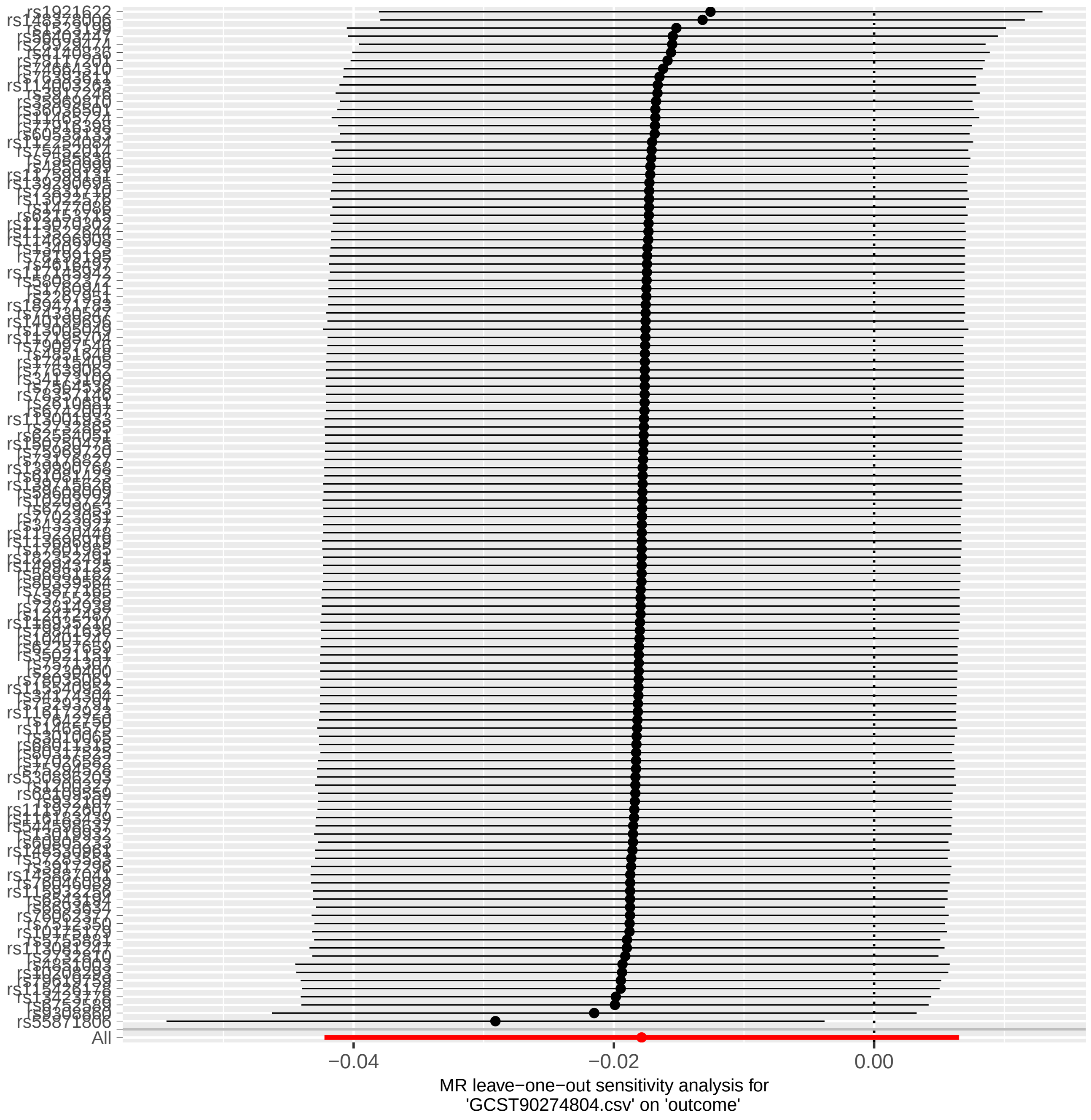
rs1921622
rs55871806
All
−0.04
−0.02
0.00
MR leave−one−out sensitivity analysis for
'GCST90274804.csv' on 'outcome'
